# Ancestral gene acquisition as the key to virulence potential in environmental *Vibrio* populations

**DOI:** 10.1038/s41396-018-0245-3

**Published:** 2018-08-02

**Authors:** Maxime Bruto, Yannick Labreuche, Adèle James, Damien Piel, Sabine Chenivesse, Bruno Petton, Martin F. Polz, Frédérique Le Roux

**Affiliations:** 1 Ifremer, Unité Physiologie Fonctionnelle des Organismes Marins, ZI de la Pointe du Diable, CS 10070, F-29280 Plouzané, France; 2 Sorbonne Universités, UPMC Paris 06, CNRS, UMR 8227, Integrative Biology of Marine Models, Station Biologique de Roscoff, CS 90074, F-29688 Roscoff Cedex, France; 3 Ifremer, LEMAR UMR 6539, 11 presqu’île du Vivier, 29840 Argenton-en-Landunvez, France; 4 Parsons Laboratory for Environmental Science and Engineering, MIT, Cambridge, MA 02139 USA

## Abstract

Diseases of marine animals caused by bacteria of the genus *Vibrio* are on the rise worldwide. Understanding the eco-evolutionary dynamics of these infectious agents is important for predicting and managing these diseases. Yet, compared to *Vibrio* infecting humans, knowledge of their role as animal pathogens is scarce. Here we ask how widespread is virulence among ecologically differentiated *Vibrio* populations, and what is the nature and frequency of virulence genes within these populations? We use a combination of population genomics and molecular genetics to assay hundreds of *Vibrio* strains for their virulence in the oyster *Crassostrea gigas*, a unique animal model that allows high-throughput infection assays. We show that within the diverse Splendidus clade, virulence represents an ancestral trait but has been lost from several populations. Two loci are necessary for virulence, the first being widely distributed across the Splendidus clade and consisting of an exported conserved protein (R5.7). The second is a MARTX toxin cluster, which only occurs within *V. splendidus* and is for the first time associated with virulence in marine invertebrates. Varying frequencies of both loci among populations indicate different selective pressures and alternative ecological roles, based on which we suggest strategies for epidemiological surveys.

## Introduction

Global change due to rising temperature and ocean acidification but also more directly due to anthropogenic influences, such as widespread use of aquaculture, is predicted to lead to a worldwide increase in *Vibrio*-associated illness of marine organisms [1–3]. This development is illustrated by recent outbreaks of disease in *Crassostrea gigas* (oyster) farms in France [4, 5], in which strains related to the Splendidus clade have been implicated [6–10]. The Splendidus clade is a large group of closely related species (e.g., *V. splendidus*, *V. crassostreae, V. tasmaniensis,* and *V. cyclitrophicus*) containing several facultatively pathogenic members that affect diverse marine organisms [11, 12]. Elevated host density due to farming is a likely factor involved in *Vibrio*-associated disease. However, it is less well understood whether the bacteria are drawn from environmental reservoirs of potential pathogens or have arisen by recent acquisition of virulence traits in response to increased host availability due to aquaculture. The latter appears to be the case in *V. crassostreae*, which we recently demonstrated to have emerged as an oyster pathogen at least partially as a result of the population-specific spread of a virulence plasmid [6]. However, collections of environmental pathogens are typically biased toward isolates from diseased animals [13, 14]. It is thus difficult to assess how broadly distributed virulence potential is among *Vibrio* spp., i.e., whether virulence is limited to few strains within populations or is a trait of the entire species. We therefore reasoned that assessment of virulence properties within populations of *Vibrio* spp. in natural environments where oysters are not farmed, could be used to determine what level of taxonomic resolution has the greatest predictive power for disease risk assessment in oyster-farming areas.

Here we determine the virulence potential of a large collection of *Vibrio* strains using specific pathogen-free (SPF) oysters as a model. The bacterial strain collection derives from a single location without intense oyster farming, on the Atlantic coast of the United States (Massachusetts, Plum Island). Extensive genetic and microhabitat characterization of these isolates has been previously reported and identified genetically defined groups that correspond to ecologically distinct habitats. This is indicative of different lifestyles (free-living or attached to different types of particulate organic matter and zoo- or phytoplankton) between these groups [15–18]. Subsequent work has found multiple lines of evidence these *Vibrio* groups represent biologically distinct populations with respect to gene flow [19, 20] and interaction with other organisms (social/behavioral networks) [21–23]. As such, these populations fit species concepts as typically used in plant and animal ecology; however, we note that they are at a finer evolutionary divergence than those traditionally used to separate species in bacterial taxonomy. To assess the virulence potential of these populations, we injected a large number of strains into juvenile SPF oysters and monitored their mortality. These animals are descendants of a pool of genitors that are produced in hatchery under highly controlled conditions to minimize the influence of genetic and environmental parameters that could affect the host sensitivity to the disease [10, 24, 25]. Moreover, these SPF oysters can be used for high-throughput experimental infections with hundreds of individual bacterial isolates [9, 10, 26]. Assaying the virulence of strains by injection has drawbacks, as it fails to capture the importance of initial infection events such as mobility and chemotaxis. However, *Vibrio* spp. related to Splendidus (both virulent and nonvirulent) naturally colonize the oyster hemolymph [6] and injection in the adductor muscle allows the direct transfer of bacteria in the hemolymph. Here screening *Vibrio* strains by injection allows the identification of traits that cause death in the animals. It is thus possible to ask whether the functional units of pathogenesis for oysters are either clones that have emerged after recent acquisition of virulence factors by horizontal gene transfer (HGT) or populations of diverse genotypes with virulence as a core function [27].

We show that the ability to trigger oyster mortality is an ancestral trait of populations within the Splendidus clade and depends on at least two loci. While in some populations all members are potentially virulent, the trait occurs intermittently in other populations and some populations have lost it altogether. The ancestrally acquired gene R5.7 is necessary for full virulence and while it has co-diversified with populations, it was lost in the non-virulent populations. Across populations, additional genes may, however, play a role in virulence. This is illustrated by the occurrence of several different types of MARTX gene clusters in *V. splendidus* for which frequency-dependent selection is suggested. The widespread occurrence of virulence genes across environmental *Vibrio* populations suggests an important biological role but their different frequency also indicates that the role is population specific.

## Materials and methods

### Genome sequencing, assembly, and annotation

In the present study, a total of 37 strains (Table S1) were sequenced (Plateforme génomique, Institut Pasteur, Paris) using the Illumina HiSeq2000 technology with ~50-fold coverage as described previously [9]. Contigs were assembled de novo using Velvet [28]. Computational prediction of coding sequences together with functional assignments was performed using the automated annotation pipeline implemented in the MicroScope platform [29]. Some gene annotations were manually curated using InterPro, FigFam, PRIAM, COGs, PsortB, TMHMM, and synteny group computation.

### Protein family identification

The proteome of each strain was first compared by performing a Blastp all-vs-all. The SiLiX software [30] was used to reconstruct protein families based on reciprocal alignment length (at least 80% of sequence length) and different thresholds of identity depending on the analysis. A threshold of 60% identity was used when comparing two populations, while a threshold of 80% was used to reconstruct families within a population.

### Phylogenetic tree construction

Species trees were reconstructed based on core genes present in a single copy. Protein sequences of each family were first aligned with Muscle, filtered using Gblocks with relaxed parameters [31] and concatenated using a custom script (available at https://github.com/mbruto/OPOPOP). Phylogenetic reconstruction was done using RAxML [32] on this concatemer using a LG model of evolution with a gamma model and default parameters. For single-marker gene trees, we aligned the sequences with Muscle and performed the phylogenetic reconstruction with RAxML using a GTR model of evolution with a gamma model and default parameters.

### Phylogenetic logistic regression analysis

The correlation between gene family presence/absence and virulence (percentage of induced mortality) was computed using the phylogenetic logistic regression implemented in the R package “phylolm” [33]. Virulence was first standardized to have a mean equal to 0 and standard deviation equal to 1, as suggested in previous work [34]. The following command was used to infer a correlation coefficient: *phyloglm(Fam* *~* *Mort, phy* *=* *Tree, data* *=* *Corr_data, method* *=* *“logistic_MPLE”)*. The variable *Corr_data* is a table containing the presence/absence of a single family in the first column (named *Fam*), while the second column contains virulence values that were standardized to have a mean of 0 and a standard deviation of 1 (named *Mort*). The *Tree* variable is the species tree of the strains considered. We used the logistic_MPLE model as it appears to be the most general one and can be applied on a wider range of data sets [33].

### Ancestral character reconstruction

Ancestral character reconstructions were performed with the version 3.31 of Mesquite (http://mesquiteproject.org) using the maximum parsimony method. The presence or absence of the Splendidus specific locus R5.7/8 was coded as 1 or 0 in a character matrix. Together with the species tree, this matrix was used as an input to reconstruct the presence or absence of the cluster on each node using the unordered parsimony model, in which acquisitions and losses have an equal cost.

### Molecular microbiology

The *Vibrio* collection used for the high-throughput infection assays (population F1–18), i.e., Plum Island collection, has been described previously [15]. The *Vibrio* strains isolated from a French oyster farming area have been described by Bruto et al. [6]. Strains and plasmids used or constructed in the present study are described in Table S2 and S3. *Vibrio* isolates were grown at 20 °C in Zobell broth or agar (4 g l^−1^ bactopeptone and 1 g l^−1^ yeast extract in artificial seawater, pH 7.6), Luria-Bertani (LB), or LB-agar (LBA) + 0.5 M NaCl. *Escherichia coli* strains were grown at 37 °C in LB or on LBA for conjugation experiments and in ZYP 5052 medium [35] for induction of protein expression in *E. coli*, strain BL21 (DE3). Chloramphenicol (Cm, 5 or 25 μg ml^−1^ for *Vibrio* and *E. coli*, respectively), carbenicillin (100 μg ml^−1^), thymidine (0.3 mM), and diaminopimelate (0.3 mM) were added as supplements when necessary. Induction of the P_bad_ promoter was achieved by the addition of 0.2% l-arabinose to the growth media, and conversely, was repressed by the addition of 1% d-glucose. Deletion of selected regions or genes was performed by allelic exchange using the pSW7848T suicide plasmid [36, 37]. To this end, two 500-bp fragments flanking the target region or gene were amplified (see primer details in Table S4), cloned into pSW7848T as previously described [9], and transferred by conjugation from *E. coli* as a donor to *Vibrio* as a recipient. Subsequently, the first and second recombinations leading to pSW7848T integration and elimination were selected on Cm/glucose and arabinose-containing media, respectively. Gene inactivation was performed by cloning 500 bp of the target gene in pSW23T [38] and selecting on Cm the suicide plasmid integration obtained by a single recombination [8]. For complementation experiments, the R5.7 gene was cloned in Apa1/Xho1 sites of the pMRB plasmid known to be stable in *Vibrio* spp. [39], resulting in a constitutive expression from a P_lac_ promoter [9]. Conjugation between *E. coli* and *Vibrio* was performed at 30 °C as described previously [36]. The BL21(DE3) strains expressing the R5.7_GFP fusion proteins were constructed using the Gibson assembly method (New England Biolabs, NEB) as previously described [40]. Briefly, the fragment encoding the green fluorescent protein (GFP) was amplified from plasmid pMRB-GFP [6] (see primer details in Table S4). R5.7_F12_ and R5.7_crass_ genes, including a flexible linker were PCR-amplified from genomic DNA of strains B8 and J2-9, respectively. The pFO4 vector, that contains a His6*-*tag at the N terminus for affinity purification, was amplified by PCR inside-out. All constructs were confirmed by sequencing prior to electrocompetent BL21(DE3) transformation.

### Purification of recombinant proteins

Recombinant R5.7_F12_ and R5.7_crass_ proteins were purified as previously described [40]. Briefly, after expression of the proteins in ZYP 5052 (20 °C, 72 h) and chemical cell lysis, the lysate was clarified at 13,865 × *g* for 45 min at 4 °C. The supernatant was loaded on a 5-ml GE HisTrap HP column, washed twice with 20 mM Na_3_PO_4_, pH 8.0, 290 mM NaCl, and 5 mM imidazole, and eluted using a gradient of 1–100% 20 mM Na_3_PO_4_, pH 8.0, 290 mM NaCl, and 1 M imidazole. Fractions containing the protein of interest were pooled, concentrated (molecular weight cutoff of 10 kDa), and loaded onto a Superdex S200 column in HEPES that contains 20 mM NaCl at 150 mM, pH 7.4. Fractions were again pooled and concentrated. The concentration was calculated from *A*_280_ and the extinction coefficient was calculated using the ProtParam tool from ExPASy (*ε*_0.1%_ of 1.04).

### Experimental determination of mortality

To determine the virulence of isolates, bacteria were grown under constant agitation at 20 °C for 24 h in Zobell media. One hundred microliters of the culture (10^8^ colony-forming units, cfu) were injected intramuscularly into oysters. Co-injections of the ΔR5.7 mutant with the recombinant purified R5.7_F12_ or R5.7_crass_ proteins were performed following the same procedure, except that the tested protein (30 μg/animal) or the corresponding volume of protein solubilization buffer (as a control) were added to the bacterial suspension 1 h before injection. The bacterial concentration was confirmed by conventional dilution plating on Zobell agar. After injection, the oysters were transferred to aquaria (20 oysters per 2.5 l of aquarium) containing 1 l of aerated 5-µm-filtered seawater at 20 °C, and kept under static conditions. Experiments were performed in duplicate at least twice, and mortality was assessed after 24 h.

## Results

### Virulence is an ancestral trait of several ecological populations

**Fig. 1:**
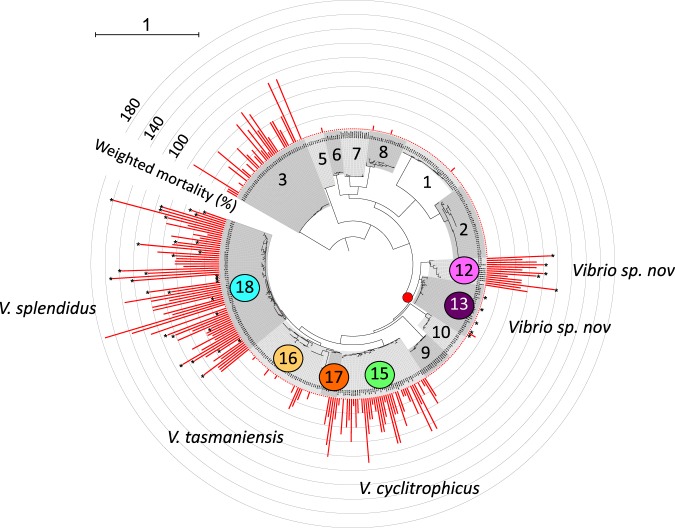
Mortality induced in oysters by *Vibrio* populations. The virulence potential mapped onto the isolate phylogeny (*Vibrio* collection from Plum Island, MA, USA). The tree is based on the genetic marker *hsp60* and comprises different genotypic clusters previously found to have a cohesive ecology and hypothesized to represent samples from natural ecological populations [15]. The closest named species to populations are as follows: F1: *Enterovibrio calviensis*; F2: *Enterovibrio norvegicus*; F3: *V. ordalii*; F4: *V. rumoiensis*; F5: *V. alginolyticus*; F7 *Aliivibrio fischeri/logei*; F8: *A. fischeri*; F9: *V. superstes*; F15: *V. cyclitrophicus*; F16–17: *V. tasmaniensis*; F18: *V. splendidus*. Populations F6, 10, 12, and 13 were not assigned to a species and were thus named here as *Vibrio sp. nov*. The node leading to the Splendidus clade in this tree is indicated by a red dot and the six populations belonging to the Splendidus clade are each indicated by distinct colors (also used in subsequent figures). Note that the presence of populations F9 and F10 within this cluster on the *hsp60* tree is an artifact of a recombination event in that gene and they are not members of the Splendidus clade, as determined by a full genome analysis. Red bars indicate weighted mortality (using *V. crassostreae* J2-9 mortality rate as a reference to evaluate variability between experiments) induced by individual strains 24 h after injection in oysters (*n* = 20). The range of variability for J2-9 between experiments (50–90%, data not shown) was lower than the variability between populations. Stars indicate the available genome sequences

We first investigated the frequency of virulence among 405 strains representing 15 ecological populations sampled randomly from Plum Island (MA) [15, 17] by injecting them individually into SPF oysters. This showed that nine populations contained exclusively non-virulent strains, while six of them were potentially virulent with the majority (5/6) of these being in the Splendidus clade (Fig. 1, populations belonging to the Splendidus clade are each indicated by distinct colours). All the strains of population F12, which represents a new species closely related to *V. crassostreae* [41], induced high levels of oyster mortality, while populations F15 (taxonomically assigned to the species *V. cyclitrophicus)*, F17 (*V. tasmaniensis*), and F18 (*V. splendidus*) are predominantly virulent, and population F16 (*V. tasmaniensis*) mostly contains non-virulent strains. The only highly virulent population outside the Splendidus clade is F3, which is taxonomically assigned to *V. ordalii*, a species that has been associated with fish disease [42]. The observed high prevalence of virulence among populations in the Splendidus clade led us to test the hypothesis that the ability to kill oysters might be an ancestral trait for the entire clade by (i) identifying putative virulence genes by comparative genomics and genetic knockout and (ii) reconstructing the acquisition and inheritance of the virulence genes among populations.

### The same genes are necessary for full virulence across the Splendidus clade

To test the hypothesis of ancestral virulence in the Splendidus clade, we first sought to identify genes potentially responsible for virulence using comparative genomics of populations F12 (*Vibrio sp. nov*) and F13 (*Vibrio sp. nov*), since these are the most closely related populations that contain exclusively virulent and non-virulent strains, respectively (Fig. 1). Using genomes of five isolates from each population, we identified 120 genes to be specific to the virulent population F12, of which 87 genes were localized in 24 genomic regions, designated regions a through x (i.e., Ra through Rx) (Table S5). Annotation implicates many of these genes in regulation, detoxification, xenobiotic degradation, siderophore production, or acquisition, i.e., the ability to respond to transient selective pressure in the environment.

**Fig. 2:**
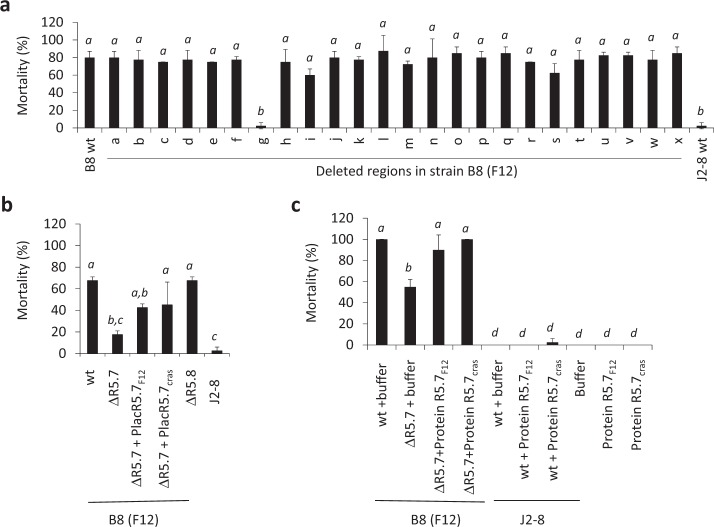
Oyster mortality in response to experimental infection with *Vibrio* wild-type strains and derivatives. **a** Twenty four population-specific regions were deleted in *Vibrio sp. nov* F12 strain B8. Virulence of the wild-type B8 strain (wt), derivatives (deletion of regions a to x), and *Vibrio sp. nov* F13-like strain J2-8 as a negative control was compared after injection of strains (10^8^ cfu/animal) into 20 oysters and counting the percent mortality at 24 h (*y* axis). **b** Comparison of mortality induced by B8 wild-type strain (wt), the mutants obtained by R5.7 or R5.8 single-gene deletion (∆R5.7 or ∆R5.8), and the complementation assay in ∆R5.7 after the transfer of a plasmid that expressed R5.7_F12_ or R5.7_cras_ constitutively from a Plac promoter (P_lac_R5.7_F12_ and P_lac_R5.7_cras_). **c** Complementation assays using recombinant purified protein R5.7, the wild-type strains (B8, J2-8) injected with buffer (wt + buffer), the mutant obtained by R5.7 single-gene deletion with buffer (∆R5.7 + buffer), the mutant ∆R5.7 or J2-8 co-injected with the recombinant purified proteins R5.7_F12_ or R5.7_cras_, the recombinant proteins, or buffer as control. All experiments were performed in duplicate and at least twice. Means with the same letter in italic are not significantly different from each other (ANOVA, *p* < 0.05 and Tukey HSD test)

Using a genetic knockout approach, we next investigated the importance of these loci for the virulence of population F12 strain 10N.286.48.B8 (hereafter named B8). None of the 24 deletions impaired bacterial growth in culture media (data not shown) and only the deletion of region Rg resulted in a decrease in oyster mortalities after experimental infection (Fig. 2a). This region contains two genes VB12B8_v1_40197 and VB12B8_v1_40198, each encoding an exported protein of unknown function. Notably, these genes showed 91 and 90% identity to the R5.7 and R5.8 proteins of *V. crassostreae*, respectively [9]. For simplicity, these genes are subsequently referred to as R5.7/8_cras_ or R5.7/8_F12_ for *V. crassostreae* and population F12, respectively. A mutant lacking the R5.7_F12_ gene (ΔR5.7_F12_) revealed that this gene contributes to B8 virulence (Fig. 2b), as previously shown for R5.7_cras_. When constitutively expressed in trans from a plasmid, R5.7_F12_ or R5.7_cras_ were sufficient to partially restore the virulence of the mutant ΔR5.7_F12_. Furthermore, a complete restoration of virulence was observed by co-injecting the ΔR5.7_F12_ with the recombinant purified R5.7_F12_ or R5.7_cras_ proteins (Fig. 2c). On the other hand, these proteins did not induce mortality when injected alone or co-injected with the non-virulent strain J2-8, showing that R5.7 is not sufficient to induce mortality. Finally, the deletion of the R5.8 gene did not alter the virulence of the B8 strain, showing that only R5.7 is necessary for the contribution of Rg to virulence.

**Fig. 3:**
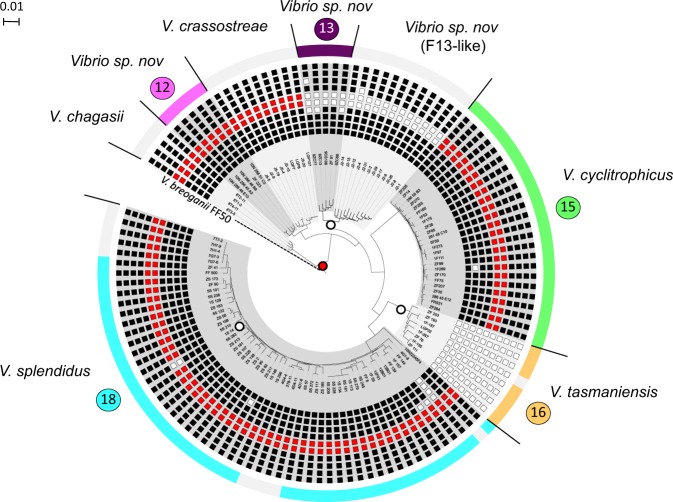
Distribution of the R5.7 and R5.8 cluster and the surrounding genes in the Splendidus clade. The phylogenetic tree was reconstructed on an alignment of 707 concatenated core proteins and rooted with *V. breoganii* FF50. The length of the outgroup branch has been arbitrarily defined (dashed line) to increase the resolution of the tree. The eight species considered are highlighted in alternating grays. Strains isolated from Plum Island are indicated by the same color code as in Fig. 1 (outer-circle). Species assignment and correspondence to population assignment in Fig. 1 are also indicated. Strains isolated from oyster-farming areas in France, i.e., *V. chagasii, V. crassostreae*, and *Vibrio sp. nov* F13-like are indicated in gray (outer-circle). The absence and presence of the R5.7/8 cluster are represented by white and red squares, respectively. The presence of the surrounding genes is indicated by a black square if they belong to the same syntenic group of the R5.7/8 gene cluster (see also Figure S1). Note that in *V. splendidus*, only the strain 1S_14 lacks the R5-7/8, while strains FF_139, FF-144, and 1S-157 lack at least one flanking gene. Red and white circles on the tree indicate acquisition and loss of R5.7/8, respectively

R5.7/8 appear highly specific to the Splendidus clade, as evidenced from the distribution in 872 available genomes from 30 different species of *Vibrio*. First, R5.7 is always colocalized with R5.8 (Fig. S1). Second, this gene cluster is restricted to the Splendidus clade encompassing the populations F12 *(Vi brio sp. nov*), F15 (*V. cyclitrophicus*), and F18 *(V. splendidus*) from the Plum Island collection, as well as populations of strains previously isolated from a French oyster farming area and assigned to *V. chagasii*, *V. splendidus,* and *V. crassostreae* [6, 9] (Fig. 3). We note that population F17 *(V. tasmaniensis)* shown in Fig. 1 from the Plum Island collection was not evaluated since there are currently no genomes available. We also confirmed the importance of the R5.7 homolog for virulence in *V. chagasii* and *V. splendidus* by inactivating this gene in strains representative of these species (Fig. S2). Finally, the genomic comparison showed that the R5.7/8 gene cluster is absent in three out of eight populations within the Splendidus clade (Fig. 3), populations F13 (*Vibrio sp. nov*) and F16 (*V. tasmaniensis*) from the Plum Island collection, and also in *Vibrio sp. nov* F13-like isolated in France. Moreover, a single non-virulent *V. splendidus* strain, 1S_14, was lacking the gene (confirmed by PCR, data not shown). Altogether, these results suggest that the R5.7 gene is necessary for full virulence across the entire Splendidus clade.

**Fig. 4:**
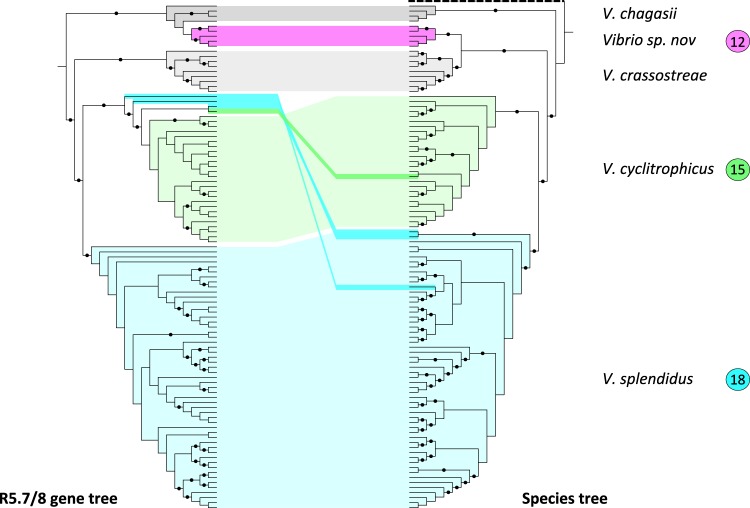
Comparison of the R5.7 and R5.8 concatenated gene tree with the species tree for populations of the Splendidus clade. The species tree (right) was reconstructed based on an alignment of 707 concatenated core proteins and rooted with *V. breoganii* FF50. Both trees are represented without branch lengths and the outgroup is represented with a dashed line on the tree. Black points on branches indicate a support value above 80% based on the rapid bootstrap analysis implemented in RAxML. Species are linked with different colors in both trees, and population correspondence with Fig. 1 is also indicated. Supported incongruences between R5.7/8 gene cluster (left) and species trees were identified for *Vibrio sp. nov* F12 (pink), one *V. cyclitrophicus* (darker green), and three *V. splendidus* strains (darker turquoise)

We next tested whether the R5.7/8 gene cluster distribution in the Splendidus clade is best explained by (i) ancestral acquisition followed by multiple losses or (ii) multiple independent acquisitions. Reconstruction of the most parsimonious ancestral character state of the R5.7/8 genes suggests an acquisition in the most recent common ancestor (MRCA) of the Splendidus clade followed by three distinct losses in the MRCAs of populations F13, F16, and F13-like (Fig. 3). The R5.7/8 gene tree is highly congruent with the species tree (Fig. 4), suggesting vertical inheritance and persistence of the gene cluster through speciation events that likely involved differential ecological specialization of the populations. The main exception to this vertical inheritance pattern is population F12, which is most closely related to *V. crassostreae* in the species tree but is sister to *V. chagasii* in the R5.7/8 tree indicating horizontal acquisition. Although the R5.7/8 gene tree clusters the vast majority of strains within their previously assigned populations, four strains are an exception: one *V. cyclitrophicus* (out of 27) and three *V. splendidus* (out of 56) appear to have acquired the R5.7/8 locus horizontally (Fig. 4). This more recent acquisition most likely involved homologous recombination since annotation of genes present within 20 kb upstream and downstream of the R5.7/8 locus did not identify any potential insertion sites for mobile genetic elements. However, such gene conversions appear to be the exception and our analysis predicts that R5.7/8 was ancestrally acquired and subsequently predominantly vertically inherited in the Splendidus clade.

### Acquisition and loss of MARTX toxin modulates virulence

The above genomic analysis also showed that R5.7 was present in non-virulent strains belonging to populations F15 and F18 (Figs. 1 and 3), suggesting that the gene is necessary but not sufficient for virulence and that other genes may play a role that these strains might have lost or never acquired. To test this hypothesis of additional virulence genes, we first computed the correlation between gene family presence/absence and induced mortalities using phylogenetic logistic regression in population F18 (*V. splendidus*) (see Materials and methods).

**Fig. 5:**
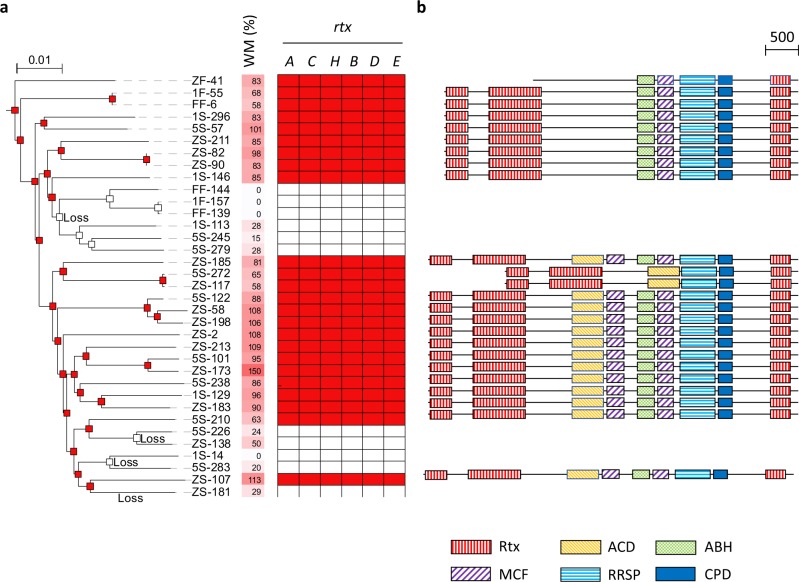
Distribution, evolutionary history, and domain variation of the *rtxACHBDE* cluster in *V. splendidus* related to induced mortality in oysters. **a** The phylogenetic tree is based on an alignment of 2290 concatenated core genes. Weighted mortalities (WM, using *V. crassostreae* J2-9 mortality rate as a reference to evaluate variability between experiments) after injection in oysters (*n* = 20) are indicated by a color gradient from white (0%) to red (150%). The range of variability for J2-9 between experiments (50–90%, data not shown) was lower than the variability between populations. Gene presence and absence is represented by red and white squares, respectively. Losses are indicated when the *rtxACHBDE* cluster is predicted to be absent on a node (or leaf) while being present in the parent node. **b** Diversity of MARTX identified in *V. splendidus*. The domain abbreviations are Rtx repeats in toxin, ACD actin cross-linking domain, ABH alpha/beta hydrolase, MCF makes caterpillars floppy, RRSP Ras/Rap1-specific endopeptidase, CPD cysteine protease domain [44]. A scale bar indicating protein length in amino acids is present on the top right of the figure

Among 12,158 families, only six genes, which were all colocalized in a single locus, showed a high correlation factor (*c* = 4.01; *P* = 0.005) (Fig. 5a). This locus encodes a putative toxin (MARTX encoded by the gene *rtxA*), a putative acyltransferase (*rtxC*), an uncharacterized protein (*rtxH*), and a putative type-I secretion system (*rtxBDE*). Because this comparison strongly suggested *rtxACHBDE* as additional virulence genes, we genetically assessed the importance of this locus for *V. splendidus* virulence.

**Fig. 6:**
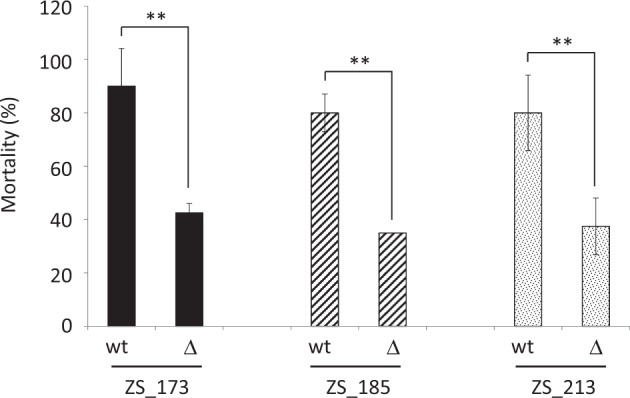
Oyster mortality in response to experimental infection with *V. splendidus* wild-type strains and ∆*rtxACHBDE* derivatives. The *rtxACHBDE* locus was deleted in three *V. splendidus* strains (ZS_173, ZS_185, and ZS_213). Virulence of the wild type (wt) and derivative (**∆**) was compared after injection of strains (10^8^ cfu/animal) in 20 oysters and counting the percentage of mortalities at 24H (*y* axis). Experiments were performed in duplicate and repeated twice. Two stars indicate highly significant differences between treatments (Wilcoxon test, *p* > 0.005)

The deletion of the complete *rtxACHBDE* locus in three strains (ZS_173, ZS_185, and ZS_213) did not impair growth in culture media, but resulted in a considerable decrease in mortalities after injection of bacteria (Fig. 6), demonstrating a role of this locus in *V. splendidus* virulence. MARTX toxins have been defined as “multifactorial effector cargo translocation, processing and delivery machines” (reviewed by refs. [43, 44]). As in other bacteria, the MARTXs found in *V. splendidus* (MARTX_Vs_) are extremely large (3646–5097 aa) and contain several domains (Fig. 5b). MARTX_Vs_ is predicted to contain the three domains found in all MARTXs, i.e., the core structure composed of two conserved regions at the amino- and carboxyl-termini and a cystein protease domain (CPD) [45]. Only one effector domain, a Ras/Rap1-specific endopeptidase (RRSP) [46, 47] is present in all MARTX_Vs_. A homolog of the actin cross-linking domain (ACD) [48] allows distinguishing two main variants of MARTX_Vs_ but the presence of ACD is not associated with heightened virulence among strains. The α/β hydrolase (ABH) and the cysteine protease (MCF) domains [49] are absent from only two *V. splendidus* strains (5S-272 and ZS-117) out of 24, that are still virulent. Finally, the Rho inactivation domain (RID) [50] found in *V. cholerae* and *V. vulnificus* and associated with a “cell rounding” effect is absent from *V. splendidus*. Hence, none of the domains ACD and RID previously demonstrated to affect polymerized actin in target eukaryotic cells, seem to be key domains of *V. splendidus* MARTX activity, while the RRSP may be hypothesized as an essential domain for its activity.

**Fig. 7:**
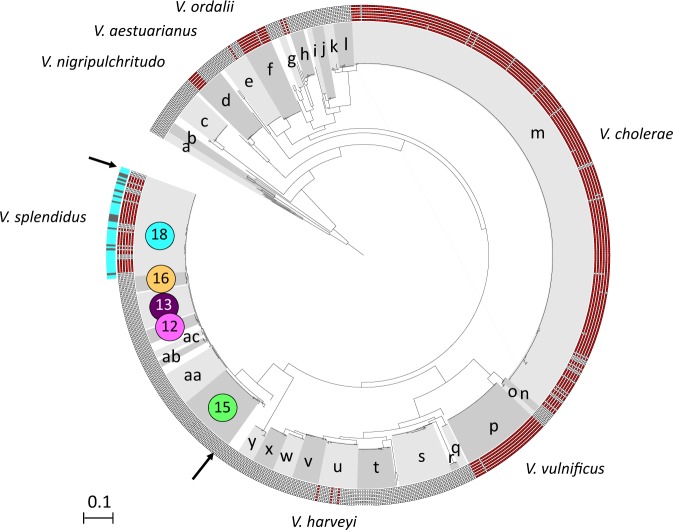
Distribution of the *rtxACHBDE* cluster in the genomes of 30 *Vibrio* species. The phylogenetic tree was reconstructed using the RctB protein sequence. The inner circle indicates the presence (red square) or absence (white square) of the six genes in the *rtxACHBDE* cluster. The outer circle indicates the isolation site of the *V. splendidus* strains with a turquoise for Plum Island and gray for oyster-farming areas in France. The 30 different *Vibrio* species and two *Vibrionaceae* genus outgroups are represented using alternating grays with the following letters: (a) *Photobacterium* spp.; (b) *Enterovibrio* spp.; (c) *V. breoganii*; (d) *V. nigripulchritudo*; (e) *V. aestuarianus*; (f) *V. ordalii*; (g) *V. scophthalmi*; (h) *V. coralliilyticus*; (i) *V. furnissii*; (j) *V. fluvialis*; (k) *V. metoecus*; (l) *V. mimicus*; (m) *V. cholerae*; (n) *V. navarrensis*; (o) *V. cidicii*; (p) *V. vulnificus*; (q) *V. natriegens*; (r) *V. parahaemolyticus*; (s) *V. alginolyticus*; (t) *V. rotiferianus*; (u) *V. harveyi*; (v) *V. campbellii*; (w) *V. owensii*; (x) *V. jasicida*; (y) *V. mediterranei*; (F15) *V. cyclitrophicus*; (aa) *V. crassostreae*; (ab) *V. chagasii*; (ac) *Vibrio sp. nov* F13-like; (F12) *Vibrio sp. nov*; (F13) *Vibrio sp. nov*; (F16) *V. tasmaniensis;* (F18) *V. splendidus*. The tree was rooted with *Photobacterium* spp. and *Enterovibrio* spp. Populations of the Splendidus clade are grouped between the two arrows and population correspondence with Fig. 1 is indicated by the same color code

In the Splendidus clade, the *rtxACHBDE* cluster is found only in *V. splendidus* but is also present in several more distantly related *Vibrio* species (Fig. 7). Within *V. splendidus*, the locus is present in 24/35 members (Fig. 5a), is chromosomally encoded, and does not show any signs of recent acquisition by HGT involving non-homologous mechanisms. Across all 872 genomes of *Vibrionaceae* (Fig. 7), this cluster is identified in seven additional species that have all been associated with disease in humans or animals [43, 44]. The frequency of the locus (strains carrying the *rtxACHBDE* cluster/total) appears different between species. In *V. cholerae, V. vulnificus*, and *V. ordalii*, the cluster is present in the vast majority of the strains, suggesting strong selection for its maintenance, irrespective of their source of isolation. In *V. cholerae* and *V. vulnificus*, 153 of 167 and 15 of 16 clinical isolates, and 37 of 43 and 16 of 16 environmental isolates contained the *rtxACHBDE* cluster, respectively. Conversely, in the shrimp pathogen *V. nigripulchritudo*, the cluster is restricted to six strains that belong to the same pathogen-containing lineage and is carried by a plasmid [51, 52]. Similarly, in *V. harveyi* and *V. aestuarianus*, the locus is detected in only 3 of 23 and 1 of 12 strains, respectively, and annotation of flanking regions indicates a recent acquisition by HGT (plasmid genes, transposase, and syntenic rupture). Hence *rtxACHBDE*, although widespread among *Vibrio* spp., appears to be present at variable frequencies within species, indicating potentially different selective pressures on the gene cluster.

## Discussion

Ecological and evolutionary dynamics of virulence genes have been poorly documented for *Vibrionaceae* in the wild, and most knowledge stems from a handful of human pathogens isolated from clinical cases where the focus has been on pathogen emergence by gene acquisition and expansion of virulent clones [53–56]. Here we show that virulence toward juvenile oysters appears to be an ancestral trait in the Splendidus clade triggered by the acquisition and subsequent vertical inheritance of the R5.7/8 gene cluster. Populations F12 (closely related to *V. crassostreae)*, F15 (*V. cyclitrophicus)*, and F18 (*V. splendidus*) have all previously been implicated in mortality of a wide range of animals [8, 57], and our results show that the ability to be virulent is characteristic of the entire populations, rather than recently emerged clones. Consequently, non-virulent populations and strains have evolved by gene loss and expansion. However, we also show that increased virulence likely involves additional gene acquisitions, as demonstrated for the *rtxACHBDE* gene cluster, which in our collection was specifically acquired by *V. splendidus*. Because our results indicate that the R5.7 gene is necessary but not sufficient for virulence, we hypothesize that the other virulent populations within the Splendidus clade (e.g., F15, *V. cyclitrophicus*) may contain additional yet-to-be-determined virulence genes.

The only virulent population outside the Splendidus clade in our collection is F3, *V. ordalii*, which has been associated with fish disease [42] and belongs to the Anguillarum clade that contains the fish pathogen *V. anguillarum* and the oyster pathogen *V. aestuarianus* [26]. This widespread distribution of pathogenicity among closely related species suggests that similar to the Splendidus clade, virulence traits might have been acquired by the MRCA of the Anguillarum clade. One candidate gene is a zinc metalloprotease, which has been demonstrated in *V. aestuarianus* to be sufficient to cause exotoxicity [58] and to be necessary for virulence in *V. anguillarum* [59]. Moreover, we also found this gene in all *V. ordalii* genomes (not shown). Hence, as is the case in the Splendidus clade, this zinc metalloprotease may be an ancestral virulence determinant gene that has been preserved through speciation events and that plays an important role in the biology of this clade.

Within the Splendidus clade, all virulent populations share the R5.7/8 gene cluster for which we show this to be ancestrally acquired. Only R5.7 seems necessary for full virulence and complementation assays using the purified R5.7_F12_ or R5.7_cras_ proteins strongly suggest that these conserved proteins interact with the external surface of *Vibrio* and/or its cellular target in oysters. The injection of the R5.7 protein alone or in the presence of non-virulent *Vibrio* strains did not affect oyster viability, showing that the protein alone does not have a lethal effect. Finally, a strong genetic linkage between R5.7/8 suggests functional interaction between these exported proteins of unknown function, in a mechanism that remains to be determined.

The R5.7/8 cluster can be present in non-virulent strains demonstrating that this cluster is necessary but not sufficient for virulence. Indeed, we show that an additional locus, the *rtxACHBDE* cluster, is necessary for full virulence towards oysters in *V. splendidus*. In other *Vibrio* species, the toxin MARTX has been demonstrated to work in concert with a cytolysin (in *V. vulnificus*) and a hemolysin (in *V. cholerae*), and it has been associated with the severity of infection by contributing to the evasion of innate immune defense [44]. An accessory role of MARTX in virulence might be expected in the shrimp pathogen *V. nigripulchritudo* [52] since it has been recently demonstrated that a lethal toxin, nigritoxin, is the major virulence factor of this pathogen [40, 51]. Among *V. splendidus* strains, MARTX contains diverse effector domains and only RRSP is shared by all strains. This domain encoding an endopeptidase has been implicated in other species in the cleavage of Ras and Rap1 proteins [46, 47]. Importantly these small GTPases play a role as regulatory nodes of intracellular signaling and membrane trafficking, including trafficking of Toll-like receptor 4 (TLR4) that is involved in innate immune response in both vertebrates and invertebrates [60]. Hence the presence and conservation of a RRSP domain suggest that in *V. splendidus*, MARTX might be involved in virulence by impairing the host innate immune responses.

The R5.7/8 and *rtxACHBDE* loci showed differential evolutionary dynamics that indicate distinct selective regimes. The high frequency within virulent populations and mostly vertical transmission of R5.7/8 suggest that it is maintained by positive selection. That three non-virulent populations within the Splendidus clade have lost the gene cluster further suggests that it is involved in niche differentiation at the species level. On the other hand, the *rtxACHBDE* cluster has been independently acquired by *V. splendidus* and distantly related other species, and this gene cluster varies in frequency across these species. Moreover, within *V. splendidus*, and similarly in *V. vulnificus* [61, 62], the composition of the effector domains appears heterogeneous, ranging from 4 to 6 effectors per toxin with only one, RRSP, found in all MARTX_vs_. In *V. vulnificus*, such compositional variation has been shown to result from homologous recombination, shuffling effector domains such that different strains within the same species can express variants of the toxins that are likely to have distinct roles in the niche [44, 61–63]. Among the diverse MARTX_vs_, 15 and 22 of 24 share an ACD and ABH domain, respectively. Moreover, 22 of 24 MARTX_vs_ share at least one MCF domain with the MARTX type III of *V. vulnificus*. Although the functional consequences of this domain variation are not fully understood, the MARTX type III of *V. vulnificus* appears to play a dual role in eel infection as well as in protection from predation by amoebae by causing the lysis of this grazer [64]. In both *V. splendidus* and *V. vulnificus*, the intermittent presence and domain diversity of the MARTX cluster are potential indicators of frequency-dependent selection [65]. Because MARTX is a very large secreted protein and hence costly to express, we hypothesize that public good dynamics may arise where producers are favored only when low in frequency within the population [66]. Additionally, variation in toxin types may arise as a result of the evolutionary arms race between bacteria and predators or host immune system and, hence, is similar to frequency-dependent selection imposed on O-antigen structure or outer membrane proteins [67–70].

Our analysis shows that a number of environmental *Vibrio* populations present a risk of virulence independent of intensive aquaculture settings. Because virulence represents a function encoded in the core genome of these populations, specific markers can be used for risk assessment rather than overall *Vibrio* abundance, as is frequently the practice. At a minimum, the R5.7/8 gene cluster may be used to indicate overall risk. However, population specific markers will facilitate a more detailed evaluation of the epidemiology of oysters. An important component of such surveys is the determination of population dynamics outside the oyster host to specifically recognize ecological conditions that may trigger population increases and hence risk of infection. Importantly, recent analysis of microbial dynamics in the coastal ocean has shown rapid, near weekly, turnover of communities that are characterized by short-lived blooms of different bacteria and eukaryotes [71]. Exploration to what extent such transient communities allow for specific blooms of different *Vibrio* populations may elucidate whether there are periods of increased risk from multiple co-occurring populations or whether risk is similar across extended periods due to sequential blooms of populations. In support of the former, we previously showed that several virulent populations of *Vibrio* can co-occur in diseased oysters, e.g., *V. crassostreae* with *V. splendidus* or *V. chagasii* [6]. We identified a plasmid that is present specifically in virulent strains of *V. crasssotreae* and necessary for full virulence [6]. Here we show that the MARTX gene cluster is involved in *V. splendidus* virulence and absent from the other virulent populations within the Splendidus clade. An interesting hypothesis to explore in the future is that other virulent populations such as *V. chagasii* or *V. cyclitrophicus* carry specific virulent traits. In a context of oyster infection by multiple co-occurring populations, these diverse virulence traits could act synergistically, increasing the vulnerability of the host. Indeed, experimental infections have demonstrated that some strains, belonging to the same [72] or different species [73], are moderately virulent when injected into oyster individually, and display heightened virulence in mixed experimental infections. Hence oyster disease may result from microbial interaction within and between populations and should be evaluated as potentially polymicrobial.

## Disclaimer

The material represents an original result and has not been submitted for publication elsewhere.

## Electronic supplementary material

Supplemental material
