# Supplementary material for: Ancestral gene acquisition as the key to virulence potential in environmental Vibrio populations
Source: ISME J. 2018 Aug 2;12(12):2954–66. doi: 10.1038/s41396-018-0245-3 (PMC6246604; doi:10.1038/s41396-018-0245-3)
Supplement: Supplementary file 1 — Supplemental material [file 41396_2018_245_MOESM1_ESM.docx]

**Supplementary Table 1**: Features of genomes sequenced in the present study

| **Population** | **Strain** | **Number of** | **Genome** | **Number of** | **Accession** |
| --- | --- | --- | --- | --- | --- |
|  | **name** | **contigs** | **size (Mb)** | **CDSs** | **number** |
| F12 | 10N.286.45.A3 | 126 | 5.48 | 5124 | PIGF00000000 |
|  | 10N.286.46.E10 | 134 | 5.39 | 5009 | PIGE00000000 |
|  | 10N.286.48.B8 | 60 | 5.23 | 4822 | PIGD00000000 |
|  | 10N.286.51.C3 | 50 | 5.19 | 4758 | PIGC00000000 |
|  | ZF 223 | 32 | 5.15 | 4727 | PIGB00000000 |
| F18 | 1F_55 | 117 | 5.74 | 5349 | PIGA00000000 |
|  | 1S_113 | 62 | 5.33 | 4842 | PIFZ00000000 |
|  | 1S_129 | 84 | 5.36 | 4875 | PIFY00000000 |
|  | 1S_14 | 41 | 5.33 | 4833 | PIFX00000000 |
|  | 1S_146 | 73 | 5.40 | 4934 | PIFW00000000 |
|  | 1S_296 | 48 | 5.40 | 4905 | PIFV00000000 |
|  | 5S_122 | 94 | 5.51 | 4998 | PIFU00000000 |
|  | 5S_210 | 41 | 5.28 | 4982 | PIFT00000000 |
|  | 5S_226 | 67 | 5.34 | 5070 | PIFS00000000 |
|  | 5S_238 | 68 | 5.41 | 4861 | PIFR00000000 |
|  | 5S_245 | 60 | 5.31 | 4781 | PIFQ00000000 |
|  | 5S_272 | 105 | 5.35 | 4853 | PIFP00000000 |
|  | 5S_279 | 69 | 5.40 | 4914 | PIFO00000000 |
|  | 5S_283 | 57 | 5.33 | 5050 | PIFN00000000 |
|  | 5S_57 | 53 | 5.41 | 5106 | PIFM00000000 |
|  | FF_139 | 196 | 5.84 | 5641 | PIFL00000000 |
|  | FF_144 | 195 | 5.90 | 5616 | PIFK00000000 |
|  | ZS_107 | 56 | 5.46 | 4920 | PIFJ00000000 |
|  | ZS_117 | 101 | 5.35 | 4847 | PIFI00000000 |
|  | ZS_138 | 90 | 5.34 | 4865 | PIFH00000000 |
|  | ZS_173 | 58 | 5.49 | 4906 | PIFG00000000 |
|  | ZS_181 | 69 | 5.28 | 4968 | PIFF00000000 |
|  | ZS_183 | 64 | 5.35 | 4841 | PIFE00000000 |
|  | ZS_185 | 102 | 5.45 | 4960 | PIFD00000000 |
|  | ZS_198 | 82 | 5.39 | 4873 | PIFC00000000 |
|  | ZS_2 | 80 | 5.47 | 4966 | PIFB00000000 |
|  | ZS_211 | 270 | 5.56 | 5282 | PIFA00000000 |
|  | ZS_213 | 96 | 5.47 | 4953 | PIEZ00000000 |
|  | ZS_58 | 78 | 5.37 | 4869 | PIEY00000000 |
|  | ZS_82 | 53 | 5.38 | 5089 | PIEX00000000 |
|  | ZS_90 | 46 | 5.38 | 5085 | PIEW00000000 |
|  | ZF_41 | 87 | 5.74 | 5310 | PIEV00000000 |

**Supplementary Table 2:** Strains used in the present study

| **Name** | **Description** | **Reference** |
| --- | --- | --- |
| PIR2 | (F-) ∆lac169 rpoS(am) robA1 creC510 hsdR514 endA recA1 uidA(∆Mlu I)::pir | Invitrogen, USA |
| β3914 | β2163 *gyrA462, zei298::Tn10* [Km^R^ Em^R^ Tc^R^] | Le Roux *et al.,* 2007 |
| BL21 (DE3) | F^-^ *ompT hsdS*_B_(r_B_^-^ m_B_^-^) *gal dcm* (DE3) | Novagen, USA |
| GV1300 | BL21 (DE3) + pFO4_GFP_R5-7_crass_ | This study |
| GV1549 | BL21 (DE3) + pFO4_GFP_R5-7_F12_ | This study |
| TOP10 | F- *mcrA Δ(mrr-hsdRMS-mcrBC) φ80lacZΔM15 ΔlacX74 recA1 araD139 Δ(ara-leu)7697 galU galK rpsL(Str^R^) endA1 nupG* | Invitrogen, USA |
| GV1460 | J2-8 (*Vibrio* sp. nov F13-like) | Lemire *et al.*, 2015 |
| GV1422 | J2-9 (*V. crassostreae*) | Lemire *et al.* 2015 |
| GV1495 | J2-9 *ΔR5.7* | Lemire *et al.*, 2015 |
| GV1040 | 10N.286.48.B8 (hereafter named B8) | Hunt *et al.*, 2008 |
| GV1826 | B8 *ΔR-a* | This study |
| GV1872 | B8 *ΔR-b* | This study |
| GV1906 | B8 *ΔR-c* | This study |
| GV1874 | B8 *ΔR-d* | This study |
| GV1895 | B8 *ΔR-e* | This study |
| GV1897 | B8 *ΔR-f* | This study |
| GV1899 | B8 *ΔR-g* | This study |
| GV1830 | B8 *ΔR-h* | This study |
| GV1601 | B8 *ΔR-i* | This study |
| GV1870 | B8 *ΔR-j* | This study |
| GV1876 | B8 *ΔR-k* | This study |
| GV1908 | B8 *ΔR-l* | This study |
| GV1832 | B8 *ΔR-m* | This study |
| GV1828 | B8 *ΔR-n* | This study |
| GV1903 | B8 *ΔR-o* | This study |
| GV1891 | B8 *ΔR-p* | This study |
| GV1887 | B8 *ΔR-q* | This study |
| GV1889 | B8 *ΔR-r* | This study |
| GV1884 | B8 *ΔR-s* | This study |
| GV1885 | B8 *ΔR-t* | This study |
| GV1858 | B8 *ΔR-u* | This study |
| GV1850 | B8 *ΔR-v* | This study |
| GV1901 | B8 *ΔR-w* | This study |
| GV1905 | B8 *ΔR-x* | This study |
| GV1832 | B8 *ΔR-m* | This study |
| GV1828 | B8 *ΔR-n* | This study |
| GV1903 | B8 *ΔR-o* | This study |
| GV1568 | B8 *ΔR5.7* | This study |
| GV2825 | B8 *ΔR5.8* | This study |
| GV2620 | B8 *ΔR-g* + pMRB-P_LAC_*R-5.7_F12_* | This study |
| GV2621 | B8 *ΔR5-7* + pMRB-P_LAC_*R-5.7_F12_* | This study |
| GV2288 | 8T2_11 (*V. chagasii, #3*) | Bruto et al., 2016 |
| GV2455 | 8T2_11 *ΔR5.7* | This study |
| GV2423 | 7T7_2 (*V. splendidus,* #24) | Bruto et al., 2016 |
| GV2493 | 7T7_2 *ΔR5.7* | This study |
| GV1054 | ZS_173 (*V. splendidus*, F18) | Hunt *et al.*, 2008 |
| GV2266 | ZS_173 *ΔMARTX* | This study |
| GV1065 | ZS_185 (*V. splendidus*, F18) | This study |
| GV1882 | ZS_185 *ΔMARTX* | This study |
| GV2466 | ZS_185 *ΔR5-7* | This study |
| GV1067 | ZS_213 (*V. splendidus*, F18) | Hunt *et al.*, 2008 |
| GV2267 | ZS_213 *ΔMARTX* | This study |

**Supplementary Table S3 :** Plasmids used and constructed in this study

| **Name** | **Description** | **Reference** |
| --- | --- | --- |
| pSW23T | *oriV*_R6Kγ_; *oriT*_RP4_; Cm^R^ | Demarre *et al.*, 2005 |
| pSWδR5.7 | pSW23T; *Δ R*5.7_8T2_11 & ZS_185_ | This study |
| pSW7848T | *oriV*_R6Kγ_; *oriT*_RP4_; *araC-*P_BAD_*ccdB;* Cm^R^ | Le Roux *et al.,* 2007 |
| pSWδR-a | pSW7848T; *Δ R-a* | This study |
| pSWδR-b | pSW7848T; *Δ R-b* | This study |
| pSWδR-c | pSW7848T; *Δ R-c* | This study |
| pSWδR-d | pSW7848T; *Δ R-d* | This study |
| pSWδR-e | pSW7848T; *Δ R-e* | This study |
| pSWδR-f | pSW7848T; *Δ R-f* | This study |
| pSWδR-g | pSW7848T; *Δ R-g* | This study |
| pSWδR-h | pSW7848T; *Δ R-h* | This study |
| pSWδR-i | pSW7848T; *Δ R-i* | This study |
| pSWδR-j | pSW7848T; *Δ R-j* | This study |
| pSWδR-k | pSW7848T; *Δ R-k* | This study |
| pSWδR-l | pSW7848T; *Δ R-l* | This study |
| pSWδR-m | pSW7848T; *Δ R-m* | This study |
| pSWδR-n | pSW7848T; *Δ R-n* | This study |
| pSWδR-o | pSW7848T; *Δ R-o* | This study |
| pSWδR-p | pSW7848T; *Δ R-p* | This study |
| pSWδR-q | pSW7848T; *Δ R-q* | This study |
| pSWδR-r | pSW7848T; *Δ R-r* | This study |
| pSWδR-s | pSW7848T; *Δ R-s* | This study |
| pSWδR-t | pSW7848T; *Δ R-t* | This study |
| pSWδR-u | pSW7848T; *Δ R-u* | This study |
| pSWδR-v | pSW7848T; *Δ R-v* | This study |
| pSWδR-w | pSW7848T; *Δ R-w* | This study |
| pSWδR-x | pSW7848T; *Δ R-x* | This study |
| pSWδR5.7_F12_ | pSW7848T; *Δ R*5.7_F12_ | This study |
| pSWδ5.8_F12_ | pSW7848T; *Δ R*5.8_F12_ | This study |
| pSWδMARTX | pSW7848T; *Δ* MARTX | This study |
| pMRB-P_LAC_*R-5.7* _F12_ | *oriV*_R6Kγ_; *oriT*_RP4_; *oriV_pB1067 ;_* P_lac_*R-5.7* _F12_ [Cm^R^] | This study |
| pMRB-P_LAC_*GFP* | *oriV*_R6Kγ_; *oriT*_RP4_; *oriV_pB1067 ;_* P_lac_*GFP* [Cm^R^] | Bruto *et al.*, 2016 |
| pFO4 | Amp^R^, T7lac promoter, His.Tag | Groisillier *et al.*, 2010 |
| pFO4-R5.7_F12_ | pFO4-HisTag-GFP-R5.7_F12_ | This study |
| pFO4-R5.7_crass_ | pFO4-HisTag-GFP-R5.7_crass_ | This study |

**Supplementary Table 4:** Primers used in this study

| **Use** | **Name** | **Sequence 5’-3’** ^a^ |
| --- | --- | --- |
| Deletion of R-a | R-a-1 | GCCC**GAATTC**GACATATCCCGGTGGTTCAC |
|  | R-a-2 | CCAATAAAGAGAGGAGCCTGGGAAGTTAGTGGGCTTTGTG |
|  | R-a-3 | CACAAAGCCCACTAACTTCCCAGGCTCCTCTCTTTATTGG |
|  | R-a-4 | GCC**CGAATTC**CAACACACCACCAACCTACG |
| Deletion of R-b | R-b-1 | GCCC**GAATTC**CATCGCCCCAGATTTACTGG |
|  | R-b-2 | CACTCGTTATGCTGGTGAGTGGAGTCGTTTGTGTAAAGGG |
|  | R-b-3 | CCCTTTACACAAACGACTCCACTCACCAGCATAACGAGTG |
|  | R-b-4 | GCCC**GAATTC**GTGTGGTTACGCCTTCATCG |
| Deletion of R-c | R-c-1 | GCCC**GAATTC**CTTGCCGCTCTCGTAGATTG |
|  | R-c-2 | CGATACTTCTGGCTGCCATAGTGAGGCATTGAGCTTTGTG |
|  | R-c-3 | CACAAAGCTCAATGCCTCACTATGGCAGCCAGAAGTATCG |
|  | R-c-4 | GCCC**GAATTC**CGTCGCGATAAAAGTAACGC |
| Deletion of R-d | R-d-1 | GCCC**GAATTC**CCGATTCATCAGAGTTCCAG |
|  | R-d-2 | CAGGGTTACTGGAATTGTCCGCAAAGGTGAGGGTTTCAGG |
|  | R-d-3 | CCTGAAACCCTCACCTTTGCGGACAATTCCAGTAACCCTG |
|  | R-d-4 | GCCC**GAATTC**GGTAACGTAAACGTGATGCG |
| Deletion of R-e | R-e-1 | GCCC**GAATTC**GGGTACTGTGTCACTGTCTC |
|  | R-e-2 | CAACTAGTGCAATGAGAGCCAAGCCAGTCAATAGACTGGC |
|  | R-e-3 | GCCAGTCTATTGACTGGCTTGGCTCTCATTGCACTAGTTG |
|  | R-e-4 | GCCC**GAATTC**CTTGGCATCGCTTCAACACC |
| Deletion of R-f | R-f-1 | GCCC**GAATTC**CCTGCATTGCTGAGTCGAAG |
|  | R-f-2 | GAGCCTGAAGCGTATCAGATCCATGCTATGTCGGCCTTTG |
|  | R-f-3 | CAAAGGCCGACATAGCATGGATCTGATACGCTTCAGGCTC |
|  | R-f-4 | GCCC**GAATTC**CAAGTTACTCTCCCACGGAG |
| Deletion of R-g | R-g-1 | GCCC**GAATTC**CTACGCAGCCAACATGCAAC |
|  | R-g-2 | GGCAAAGGCATTTTCACCGTGCCATAAAAAGGCCGACATC |
|  | R-g-3 | GATGTCGGCCTTTTTATGGCACGGTGAAAATGCCTTTGCC |
|  | R-g-4 | GCCC**GAATTC**TTCGATCTCGATTGGACTGC |
| Deletion of R-h | R-h-1 | GCCC**GAATTC**GTGATCCAGCCTTTGTTCAG |
|  | R-h-2 | GATATTCGCGAAGCAGCACCCCTGTTGAGTTGACAGGAGG |
|  | R-h-3 | CCTCCTGTCAACTCAACAGGGGTGCTGCTTCGCGAATATC |
|  | R-h-4 | GCCC**GAATTC**CTTTGGAGCTGCGTTCAAAC |
| Deletion of R-i | R-i-1 | GCCC**GAATTC**GGTAGTGGCTGATGAAGTTC |
|  | R-i-2 | GCTGCCGTGGTGATATGTATAAAATGTCCCGCATTAGGCG |
|  | R-i-3 | CGCCTAATGCGGGACATTTTATACATATCACCACGGCAGC |
|  | R-i-4 | GCCC**GAATTC**TGGCGCTGTATGACCAAAAC |
| Deletion of R-j | R-j-1 | GCCC**GAATTC**GCAGAAGTATGGTAAGTCCC |
|  | R-j-2 | GAAGTACCCAGGGTCGTTTGAATGCCAATACCAAGCGCAG |
|  | R-j-3 | CTGCGCTTGGTATTGGCATTCAAACGACCCTGGGTACTTC |
|  | R-j-4 | GCCC**GAATTC**CATGCTTTAGGTGCACACGC |
| Deletion of R-k | R-k-1 | GCCC**GAATTC**AGACACAGACATCGGCTATG |
|  | R-k-2 | GCATTGAAGTAGGGGACATCGTGAGCAAATAGCGTACCTG |
|  | R-k-3 | CAGGTACGCTATTTGCTCACGATGTCCCCTACTTCAATGC |
|  | R-k-4 | GCCC**GAATTC**CGTATTGGTGATGTAGCCTC |
| Deletion of R-l | R-l-1 | GCCC**GAATTC**CCAGATAACGTAGCAGCAGG |
|  | R-l-2 | GTGCCTACAGTCCGAGTAATAAACAGCCAGCATAAGGCTG |
|  | R-l-3 | CAGCCTTATGCTGGCTGTTTATTACTCGGACTGTAGGCAC |
|  | R-l-4 | GCCC**GAATTC**CTGCGCTGTCGTTAGCAAAG |
| Deletion of R-m | R-m-1 | GCCC**GAATTC**ACCAACCAACGAAAGTGGTG |
|  | R-m-2 | CAAGATGTTACCTCTGCGCTGTGTTGCAGCTTTCATTGGC |
|  | R-m-3 | GCCAATGAAAGCTGCAACACAGCGCAGAGGTAACATCTTG |
|  | R-m-4 | GCCC**GAATTC**CACTTCCATAACCTCTGTGC |
| Deletion of R-n | R-n-1 | GCCC**GAATTC**CCAAGACACAATCCATCACC |
|  | R-n-2 | CAGTGGTGAAGTTCAGAAGCTATTAGGTGACCTCCCAACG |
|  | R-n-3 | CGTTGGGAGGTCACCTAATAGCTTCTGAACTTCACCACTG |
|  | R-n-4 | GCCC**GAATTC**CGACATCGAGAAACTTGGTC |
| Deletion of R-o | R-o-1 | GCCC**GAATTC**GTTGCGTTTGCGGTATCAAG |
|  | R-o-2 | CCCGAAGGCTACACTTATTCGACTCTGTGAGCATGTTTGG |
|  | R-o-3 | CCAAACATGCTCACAGAGTCGAATAAGTGTAGCCTTCGGG |
|  | R-o-4 | GCCC**GAATTC**ATAGTCTATCGTGCTGCAGG |
| Deletion of R-p | R-p-1 | GCCC**GAATTC**CCTAATTCAGGATCGCCATC |
|  | R-p-2 | CATACGCTCAGCAGTATTCGCAAGCACGATGACAGTTGAC |
|  | R-p-3 | GTCAACTGTCATCGTGCTTGCGAATACTGCTGAGCGTATG |
|  | R-p-4 | GCCC**GAATTC**GGTCAGATCAAAGACCGATG |
| Deletion of R-q | R-q-1 | GCCC**GAATTC**GAGACTTCGTGTGTCTCTGC |
|  | R-q-2 | GTGGGCTTTTTAACGTCTGCCTGGTACATTAGCCAGCTTC |
|  | R-q-3 | GAAGCTGGCTAATGTACCAGGCAGACGTTAAAAAGCCCAC |
|  | R-q-4 | GCCC**GAATTC**GCAATGGAAGAGCAAGACAG |
| Deletion of R-r | R-r-1 | GCCC**GAATTC**TTCCTGAACAGTAAGCGGAC |
|  | R-r-2 | GAACATCGATTCTCAGCCTCAATTGGGGAGAGCATTAGGC |
|  | R-r-3 | GCCTAATGCTCTCCCCAATTGAGGCTGAGAATCGATGTTC |
|  | R-r-4 | GCCC**GAATTC**GGTAGGATGGATGAGGAAAC |
| Deletion of R-s | R-s-1 | GCCC**GAATTC**GCATCGAACAATGGGATTGG |
|  | R-s-2 | CGGTATTACCCAACGGAAACGACATAGAGTAAGACCTCGC |
|  | R-s-3 | GCGAGGTCTTACTCTATGTCGTTTCCGTTGGGTAATACCG |
|  | R-s-4 | GCCC**GAATTC**CTACTTCTCGGCCATCAAAC |
| Deletion of R-t | R-t-1 | GCCC**GAATTC**CTAAACAGTACTCAGCGCTC |
|  | R-t-2 | CTCGTATCTAGCTACCCTTCGTAAGCAAGCCAAGCATCAG |
|  | R-t-3 | CTGATGCTTGGCTTGCTTACGAAGGGTAGCTAGATACGAG |
|  | R-t-4 | GCCC**GAATTC**GTTCCTACGTGAACTACCAG |
| Deletion of R-u | R-u-1 | GCCC**GAATTC**AAGGCTATCGTGCCGCATTC |
|  | R-u-2 | CCAATACTGCCACAAAGTGCATTTGTGCGGACAACCTACG |
|  | R-u-3 | CGTAGGTTGTCCGCACAAATGCACTTTGTGGCAGTATTGG |
|  | R-u-4 | GCCC**GAATTC**CCTGAGGTTGACCACGTTCG |
| Deletion of R-v | R-v-1 | GCCC**GAATTC**TGAACAAGCGTGTGATCACC |
|  | R-v-2 | GGTAAGGGGTCGACTTTACCGCTCACGCTAAAGTAACCAC |
|  | R-v-3 | GTGGTTACTTTAGCGTGAGCGGTAAAGTCGACCCCTTACC |
|  | R-v-4 | GCCC**GAATTC**GCACACTATCGACATGACAG |
| Deletion of R-w | R-w-1 | GCCC**GAATTC**CACAATGGACAAGTCCCATC |
|  | R-w-2 | GACACTTGTCAGCCAAACACCTTCCATGCCCAAACTGGAG |
|  | R-w-3 | CTCCAGTTTGGGCATGGAAGGTGTTTGGCTGACAAGTGTC |
|  | R-w-4 | GCCC**GAATTC**GCACCGCTAAGTCATCAACC |
| Deletion of R-x | R-x-1 | GCCC**GAATTC**CAAGTAATGCGGGACTGGAC |
|  | R-x-2 | CAACCCCAGTTCTCAACAGGGATGTTGCAACGTTACCGAC |
|  | R-x-3 | GTCGGTAACGTTGCAACATCCCTGTTGAGAACTGGGGTTG |
|  | R-x-4 | GCCC**GAATTC**ATGTTCAAGTGCCTCACCTC |
| Deletion of R5.7 | R5.7-1 | GTATCGATAAGCTTGATATC**GAATTC**CTCTTACCATCGCTCTTCAG |
|  | R5.7-2 | CCAAAAAGGCCGACAAGATGGGGTAGCAGAAACCAACACC |
|  | R5.7-3 | GGTGTTGGTTTCTGCTACCCCATCTTGTCGGCCTTTTTGG |
|  | R5.7-4 | CCCCCGGGCTGCAG**GAATTC**CTACGCAGCCAACATGCAAC |
| Deletion of R5.8 | R5.8-1 | GTATCGATAAGCTTGATATC**GAATTC**TCTCCTGCAATCACAGTCAC |
|  | R5.8-2 | CTAATCACAAATCTAACAGGGATAAAACTCCATACCCATCGACAAATAG |
|  | R5.8-3 | CTATTTGTCGATGGGTATGGAGTTTTATCCCTGTTAGATTTGTGATTAG |
|  | R5.8-4 | CCCCCGGGCTGCAG**GAATTC**ACCTCTTAGTTATTTGGCACAG |
| Inactivation of R5.7 | 0504-1 | CGTATGCCTGAACATAGTTAG |
|  | 0504-2 | GGGATCTGATGATCACCGAG |
| Deletion of *rtxACHBDE* | MX1 | GCCC**GAATTC**AGATACATCGGACCATGCTG |
|  | MX2 | GGATACTCAGGTACAGCCTAAGGAAGTGACGTGATGGTAC |
|  | MX3 | GTACCATCACGTCACTTCCTTAGGCTGTACCTGAGTATCC |
|  | MX4 | GCCC**GAATTC**GCTCGAATTGATAGGTTGCG |
| R5.7_F12_ in MRB | 1102-1 | GTGAGCGGATAACAAAGGAA**GGGCCC**ATGAGAATTCTACCGATTATTATTTCTC |
|  | 1102-2 | CGACGCGTCTGCAG**CTCGAG**TCAGTTAGAACAACTGTTGCCATTC |
|  | 0207-1 | **CTCGAG**CTGCAGACGCGTCG |
|  | 0207-2 | **GGGCCC**TTCCTTTGTTATCCGCTCAC |
| R5.7_F12_  in pFO4 | 1102-3 | GGTGGTGGTTCTGGTGGTGGTTCTAGTGACTGGCTAGAGCTTAATAAC |
|  | 1102-4 | CATGCATGCTAGCCTCGAGTTAGTTAGAACAACTGTTGCCATTC |
|  | 0904-1 | CATCACCATCACCATGGATCCAGTAAAGGAGAAGAACTTTTC |
|  | 0904-2 | AGAACCACCACCAGAACCACCACCTTTGTATAGTTCATCCATGC |
|  | 2003-1 | GGA TCC ATG GTG ATG GTG ATG |
|  | 2003-2 | CTC GAG GCT AGC ATG CAT G |
| R5.7_cras_ in pFO4 | 0904-5 | GGTGGTGGTTCTGGTGGTGGTTCTAGCGACTGGCTTGAACTG |
|  | 0904-6 | CATGCATGCTAGCCTCGAGTTAATTAGAGCAGCTATTGCC |

^a^ restriction sites are indicated in bold

**Supplementary table 5**: Annotation of *Vibrio* sp. F12 specific regions.

| **Region** | **Locus tag^a^** | **Product** |
| --- | --- | --- |
| R-a | 160071 | AraC-type DNA-binding domain-containing protein |
|  | 160070 | Putative MOSC domain protein |
| R-b | 180051 | Iron-hydroxamate transporter permease subunit |
|  | 180052 | Ferrichrome-binding protein |
|  | 180053 | Ferrichrome outer membrane transporter |
|  | 180055 | Transcriptional activator |
|  | 180056 | Conserved protein of unknown function with ferric iron reductase domains |
|  | 180057 | Iron-hydroxamate transporter subunit ; ATP-binding component of ABC superfamily |
| R-c | 10026 | Putative Transcription regulator, AsnC-type protein |
|  | 10027 | Conserved membrane protein of unknown function |
| R-d | 10384 | Putative Transcription regulator, TetR-like |
|  | 10385 | Putative dihydrofolate reductase |
|  | 10386 | Proton/sodium-glutamate symport protein |
| R-e | 40063 | NADH:flavorubredoxin oxidoreductase |
|  | 40065 | Flavorubredoxin oxidoreductase |
| R-f | 40179 | Transcriptional regulator, LysR family protein |
|  | 40180 | Conserved exported protein of unknown function |
| R-g | 40197 | Conserved exported protein of unknown function (R5.7) |
|  | 40198 | Conserved exported protein of unknown function (R5.8) |
| R-h | 40249 | Conserved membrane protein of unknown function |
|  | 40250 | Uncharacterized HTH-type transcriptional regulator |
|  | 40251 | Putative glutathione S-transferase |
|  | 40252 | Putative transcriptional regulator, LysR family protein |
|  | 40253 | Putative methyl-accepting chemotaxis protein |
|  | 40254 | Cryptochrome-like protein cry2 |
|  | 40255 | Sigma-54 dependent transcriptional regulator |
|  | 40256 | Aldehyde dehydrogenase B |
|  | 40257 | Putative NUDIX hydrolase |
|  | 40258 | Conserved protein of unknown function |
|  | 40259 | Putative multidrug efflux transporter |
|  | 40260 | S-formylglutathione hydrolase |
|  | 40261 | Alcohol dehydrogenase class 3 (Alcohol dehydrogenase class III) |
|  | 40262 | Quaternary ammonium compound-resistance protein QacE |
|  | 200002 | Murein peptide amidase A |
|  | 200003 | Periplasmic oligopeptide-binding protein |
|  | 200004 | Putative Ethylbenzene dehydrogenase |
|  | 200006 | Putative cytochrome c-type protein NrfB |
|  | 200007 | Putative tetraheme cytochrome c3 |
| R-i | 200027 | Putative Organic hydroperoxide resistance protein |
|  | 200028 | Putative Organic hydroperoxide resistance transcriptional regulator |
| R-j | 200054 | Putative two component histidine kinase |
|  | 200055 | Two-component response regulator of C4-dicarboxylate transport |
|  | 200056 | Putative glycerophosphoryl diester phosphodiesterase |
|  | 200057 | Putative Phosphonate-binding periplasmic transporter |
|  | 200058 | Putative Phosphonates import ATP-binding protein PhnC |
|  | 200059 | Putative PhnE phosphonate ABC transporter, permease |
|  | 200060 | Putative Phosphonate ABC transporter permease protein phnE1 |
| R-k | 310012 | Conserved protein of unknown function |
|  | 310011 | Conserved exported protein of unknown function |
| R-l | 130064 | Putative 5'-nucleotidase |
|  | 130062 | Multiple antibiotic resistance protein marC |
| R-m | 100119 | Putative membrane bound peptidase; NefD homolog |
|  | 100118 | Putative Stomatin like transmembrane protein |
| R-n | 100095 | Conserved exported protein of unknown function |
|  | 100094 | Conserved protein of unknown function |
| R-o | 30035 | Putative Signal transduction protein containing an EAL and a GGDEF domains |
|  | 30034 | Putative ABC-type sugar transport system, periplasmic component |
|  | 30033 | Putative Ribose ABC transporter, periplasmic ribose-binding protein |
| R-p | 170092 | Conserved exported protein of unknown function |
|  | 170091 | Conserved exported protein of unknown function |
|  | 170090 | Conserved protein of unknown function |
|  | 170089 | Conserved exported protein of unknown function |
|  | 170088 | Conserved exported protein of unknown function |
|  | 170087 | Putative Transcriptional regulator, LuxR family |
|  | 170081 | Endonuclease/Exonuclease/phosphatase family protein |
| R-q | 80118 | GGDEF family protein |
|  | 80117 | Putative Outer membrane protein and related peptidoglycan-associated (lipo)proteins |
|  | 80116 | Conserved protein of unknown function |
|  | 80115 | Conserved protein of unknown function |
|  | 80114 | TadE-like protein |
|  | 80113 | Conserved exported protein of unknown function |
|  | 80112 | Conserved membrane protein of unknown function |
|  | 80111 | Putative Flp pilus assembly protein TadB |
|  | 80109 | Putative Flp pilus assembly ATPase TadZ/CpaE |
|  | 80108 | Conserved exported protein of unknown function |
|  | 80106 | Putative Flp pilus assembly protein RcpC/CpaB |
|  | 80105 | Conserved membrane protein of unknown function |
|  | 80104 | Putative pilin Flp |
|  | 80103 | Bacterial regulatory helix-turn-helix , lysR family protein |
| R-r | 80026 | Conserved exported protein of unknown function |
|  | 80025 | Conserved exported protein of unknown function |
|  | 80024 | Conserved protein of unknown function |
|  | 80023 | Conserved exported protein of unknown function |
| R-s | 80012 | Fe2+/Zn2+ uptake regulation protein |
|  | 80011 | Adenine deaminase family protein |
|  | 80010 | Putative carbonate dehydratase |
|  | 80009 | Putative Cobalamin (vitamin B12) biosynthesis CobW-like |
| R-t | 250021 | Transcriptional regulator |
|  | 250022 | Conserved membrane protein of unknown function |
| R-u | 60205 | Conserved exported protein of unknown function |
|  | 60204 | Conserved protein of unknown function |
| R-v | 60093 | Putative Cytochrome c556 |
|  | 60092 | Hydrogenase cytochrome B-type subunit-like protein |
|  | 60091 | Transcriptional regulator, MarR family protein |
| R-w | 60041 | Transcriptional regulator |
|  | 60040 | Conserved exported protein of unknown function |
| R-x | 350020 | Predicted Na+/dicarboxylate symporter |
|  | 440001 | Coenzyme A disulfide reductase |
|  | 440002 | Conserved exported protein of unknown function |

^a^Locus tag in B8 (VB12B8_v1_XXXXXX)

**Supplementary Figure 1:** **Comparison of the genetic organization of the R5-7/8 locus in the Splendidus clade.** The first genetic organization (top of the figure) is mostly found in the populations of the Splendidus clade and was used as a reference for Figure 3. The R5.7 and R5.8 genes are represented with red arrows. The surrounding genes of R5-7/8 are represented by black arrows shrouded by colors to keep track in the different genetic organization shown in this figure. Genes in grey are intermittently present in the R5-7 locus and encode for different metabolic activities that are indicated when known. The distribution among different species the Splendidus clade of the different genetic organization of the R5-7/8 locus is shown on the right.

**Supplementary Figure 2:** **Virulence of R5.7 inactivated strains belonging to the Splendidus clade.** Comparison of mortalities induced by wild type strain (wt) and R5.7 inactivated mutant of a strain representative of *V. chagasii* (8T2_11) and *V. splendidus* (7T7_2) isolated in France (population #3 and 24 in Bruto et al., 2016) and *V. splendidus* isolated in Plum Island (population F18 in Hunt et al., 2008)*.* All infections were performed in duplicate and at least twice by intramuscular injection of strains (10^7^ cfu/animal) in 20 oysters and counting the percentage of mortalities at 24H (y-axis).
